# Intrapulmonary administration of recombinant activated factor VII in diffuse alveolar haemorrhage: a report of two case stories

**DOI:** 10.1186/1757-1626-1-150

**Published:** 2008-09-12

**Authors:** Ángel Estella, Antonio Jareño, Luis Perez-Bello Fontaiña

**Affiliations:** 1 Critical Care Unit. Hospital of Jerez. Spain

## Abstract

**Background:**

Diffuse alveolar haemorrhage (DAH) is a serious pulmonary complication characterised by a high mortality rate and the absence of specific treatment. The intrapulmonary administration of activated recombinant factor VII (rFVIIa) in DAH was recently published in six patients by Heslet et al with an efficient hemostatic effect. We describe two cases of DAH treated with intrapulmonary rFVIIa.

**Methods:**

Two cases of DAH were admitted to the ICU after presenting abrupt desaturation, tachypnea, cough and haemoptysis, requiring orotracheal intubation and mechanical ventilation. The diagnosis was achieved by the bloody return during the bronchoalveolar lavage, during the procedure rFVIIa (50 μg/Kg in 50 ml of isotonic saline) was administered via the bronchoscope.

**Results:**

Immediate cessation of bleeding was observed. Prior to intrapulmonary administration of rFVIIa, the FiO_2 _was 1, which was reduced to 0.4 24 hours later. Following the procedure, the haemostatic effect made blood transfusion superfluous. No thrombotic complications associated with administration of the drug were observed. After the intervention both cases progressed fast and was discharged from the ICU with no further episodes of bleeding.

**Conclusion:**

1. Local intrabronchial deposition of DAH with rFVIIa has been shown to be effective in controlling life-threatening DAH. 2. In the case described above, no thrombotic complications were observed following the intrapulmonary administration of rFVIIa.

## Background

Diffuse alveolar haemorrhage (DAH) is a serious pulmonary complication, characterised by the presence of haemoptysis, dyspnea, hypoxemia and anaemia, with a high mortality rate of over 50% of patients requiring mechanical ventilation [1]. Diffuse opacities found on X rays in patients with DAH, however, are unspecific [2].

DAH is a complication of systemic diseases, and frequently manifests as an initial sign of these [3]. Bronchoalveolar lavage (BAL) is the most useful procedure for confirming initial clinical suspicion, BAL with a bloody return is the only way to confirm the diagnosis and, at times, fiberoptic bronchoscopy provides the treatment. In a new development, its use in intrapulmonary administration of recombinant activated factor VII (rFVIIa) has been reported in six patients [4].

The drug (rFVIIa) promotes local formation of thrombin when it combines with tissue factor exposed at the level of the endothelium. It was initially indicated to treat coagulopathies in patients suffering from haemophilia, although its use has extended to other haematological conditions. Beyond these indications, it is administrated on a more compassionate level. The requirements for its effective use in controlling serious haemorrhages are fibrinogen and platelet readings of over a 50 mg/dl and with 50.000/μl respectively, in addition to a pH > 7.20.

We present two cases of massive haemoptysis in which local administration of rFVIIa (Novoseven^® ^from Novonordisk) via BAL was used as an emergency measure.

## Case presentation

Two cases of life-threatening DAH were admitted to the ICU after presenting abrupt desaturation, tachypnea, cough and haemoptysis, requiring orotracheal intubation and mechanical ventilation. Both cases were diagnosed with fiberoptic bronchoscopy and treated with local rFVIIa.

### Clinical case 1

A 39 year old woman, with a personal history of acute promyelocytic leukaemia treated with chemotherapy and renal failure with recent arteriovenous fistula intervention. The patient was admitted to the ICU with hypoxemic respiratory failure with cough and haemoptysis, requiring orotracheal intubation and mechanical ventilation. The thorax X-ray showed a "patchy" infiltrate affecting bases and middle fields. Values of haemoglobin with 11,4 decreased to 7,9 g/dl concomitant with reduced platelet count from 100 × 10[3]/μL to 40 × 10[3]/μL during the first 24 hours. Suddenly, the patient deteriorated with an abrupt desaturation and frank haemoptysis through the orotracheal tube. In order to achieve platelet readings of over 50,000/μL a transfusion of 8 units of platelets was necessary before the drug could be administered. An emergency fibrobronchoscopy confirmed DAH. Systemic administration of rFVIIa was considered, but the potential thrombogenic effect of the drug and the risk of obstruction of the recent arteriovenous fistula prompted us to decide to administer 50 μg/Kg of rFVIIa in 50 ml of isotonic saline via the bronchoscopy channel, 25 ml in each main bronchus; following this, immediate cessation of bleeding was observed. Prior to this, the families had been informed and their written consent obtained.

**Figure 1:**
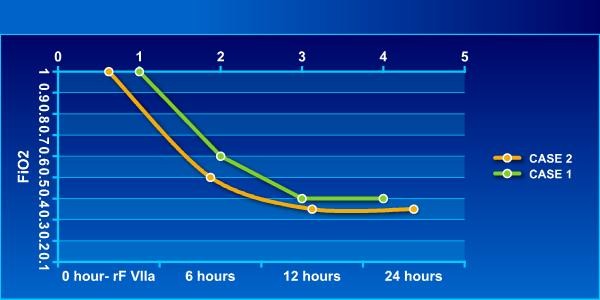
Changes in the FiO_2 _following rFVIIa administration in two cases treated with intrapulmonary rFVIIa.

The inspired fraction of oxygen (FiO_2_) which, before intrapulmonary administration of rFVIIa had been 1, was reduced to 0.6 during the first three hours subsequent to the administration of rFVIIa, and to 0.4 over the following 24 hours (figure 1). Haemoglobin readings remained unchanged without the need for blood transfusion. Although there were no new episodes of active DAH, weaning from ventilator was retarded due to muscular weakness. The patient was extubated at day 16 in the ICU and discharged to stationary ward without recurrent bleeding.

### Clinical case 2

A 46 year old man, with a personal history of smoking, ex parenteral drug abuse, hepatitis B and C infection, hepatic cirrhosis evolving for years and HIV infection diagnosed in 1988; currently receiving antiretroviral treatment. He was admitted to the ICU with acute inferoposterior myocardial infarction. Treatment was commenced with low molecular weight heparin and double anti-aggregation therapy with aspirin and clopidogrel. After 24 hours, he suddenly developed haemoptysis, acute hypoxemic respiratory failure and bilateral crackles, requiring orotracheal intubation and mechanical ventilation with FiO_2 _of 1 to maintain a SpO_2 _of 85–90%. The BAL return was increasingly bloody from both lungs. Due to the potential thrombogenic effect of systemic rFVIIa administration in acute myocardial infarction, and after informing and obtaining consent from the family, we decided on intrapulmonary administration of the drug at a dosage of 50 μg/Kg in 50 ml of isotonic saline via the bronchoscopy channel, observing the immediate cessation of bleeding. The FiO_2 _was reduced to 0.5 over the first three hours and to 0.35 after 24 hours (figure 1). After 12 days on mechanical ventilation the patient was extubated and transferred to a stationary ward.

## Discussion

Systemic administration of rFVIIa to patients with life-threatening conditions due to active haemorrhage has increased in clinical practise, based more on presumed expectation than on scientific evidence supported by controlled and randomised studies [5].

Edward et al carried out a survey in the American College of Chest Physicians (ACCP) in 1998 on the treatment of acute haemoptysis: 85% of specialists answered that intubation and connection to mechanical ventilation must be performed at an early stage and 64% considered it mandatory to carry out a fibrobronchoscopy during the first 24 hours [6]. Consequently, treatment undertaken in both clinical cases presented – mechanical ventilation support and fibrobronchoscopy – meets these recommendations and was performed as an emergency measure to treat a massive haemoptysis episode. In middle-sized hospital, where selective embolization of the bronchial artery techniques and/or thoracic surgery are not available, systemic rFVIIa has been used in massive haemoptysis cases, with good results [7].

DAH in haematological patients requiring mechanical ventilation has a high mortality rate of over 70% in series described [8,9]. This is due to the absence of specific treatment for DAH of haematological origin. The results of a multicentre, randomised study on the efficacy and safety of three different dosages of systemic rFVIIa compared with a placebo in treating haemorrhagic complications in 100 bone-marrow transplant patients (seven with DAH) were inconclusive, 8% of thromboembolic events were observed in the group treated with rFVIIa [10].

Pulmonary haemorrhage associated with myocardial infarction thrombolysis is an unusual complication; in 1996, Chang YC et al published a retrospective study, finding an incidence of 0.4% [11]. The same is true of platelet antiaggregation treatment, where isolated cases of DAH have been described [12]. In spite of its low incidence, DAH secondary to acute coronary syndrome treatment is a complication which maybe undetected, due to the common radiological findings in both DAH and ALI/ARDS and acute pulmonary oedema [13]. Bearing in mind the thrombogenic risk of a systemic administration of local rFVIIa, and with Heslet et al's publication as a reference, in which 6 consecutive critically ill patients with acute DAH are treated with local rFVIIa, intrapulmonary administration of the drug was chosen in the cases here presented with a view to avoiding the risk of thrombosis of the arteriovenous fistula in the first case, and of reinfarction in the second [4,10].

## Conclusion

DAH is a life-threatening disease characterised by the lack of specific treatment and a high mortality of patients requiring mechanical ventilation. Bronchoscopy BAL with increasingly bloody return is the only diagnostic procedure for the diagnosis of DAH, and at times, provides the treatment. Local administration of rFVIIa via the fibrobronchoscope channel was used as an emergency measure in two cases of massive haemoptysis with an excellent hemostatic effect and without adverse effects.

## Abbreviations

ALI: Acute lung injury; ARDS: Acute respiratory distress syndrome; BAL: Bronchoalveolar lavage; DAH: Diffuse alveolar haemorrhage; FiO_2_: Inspired fraction of oxygen; rFVIIa: Recombinant activated factor VII; HIV: Human inmunodeficiency virus; SpO_2_: Pulse oxygen saturation.

## Competing interests

The authors declare that they have not competing interests.

## Authors' contributions

All authors participated in the interpretation and discussion of results. AE and AJ drafted and revised the manuscript. All authors read and approved the final manuscript.

## Consent

The families were informed and their written consent obtained.
